# Why are some people more successful at lifestyle change than others? Factors associated with successful weight loss in the BeWEL randomised controlled trial of adults at risk of colorectal cancer

**DOI:** 10.1186/s12966-015-0240-2

**Published:** 2015-06-26

**Authors:** Martine Stead, Angela M. Craigie, Maureen Macleod, Jennifer McKell, Stephen Caswell, Robert J. C. Steele, Annie S. Anderson

**Affiliations:** Institute for Social Marketing, University of Stirling, Stirling, FK9 4LA Scotland UK; Centre for Research into Cancer Prevention and Screening, Cancer Division, Medical Research Institute, Level 7, Ninewells Hospital and Medical School, Dundee, DD1 9SY UK

**Keywords:** Lifestyle change, Intervention, Diet, Physical activity, Weight loss, Colorectal cancer screening, Factors, Qualitative, Quantitative

## Abstract

**Background:**

The BeWEL (**B**ody**WE**ight and physica**L** activity) randomised controlled trial demonstrated that a weight management programme offered in the colorectal cancer screening setting was effective. However, the differential responses of participants to the programme were notable. This study aimed to explore the factors associated with success and to identify implications for future programme design.

**Methods:**

Analyses were conducted of quantitative data (*n* = 148) from the BeWEL intervention group to compare demographic and psychosocial characteristics and lifestyle changes in those who met and exceeded the target 7 % weight loss (‘super-achievers’) with those who achieved only ‘moderate’ or ‘low’ amounts of weight loss (2–7 % loss, or <2 % loss, respectively). In-depth qualitative interviews (*n* = 24) explored in detail the motivations, actions, pathways to weight loss and circumstances of study participants.

**Results:**

Over the 12 month intervention period, mean percentage weight loss of super-achievers (*n* = 33) was 11.5 %, compared with moderate-achievers (*n* = 58) who lost 4.2 %, and low-achievers (*n* = 57) who gained 0.8 %. Compared to other groups, super- achievers increased their fruit and vegetable intake (*p* < 0.01) and physical activity (step count, *p* < 0.01). ‘Super-achievers’ did not differ in baseline demographic characteristics from other participants. However, significantly fewer reported that their activities were limited by physical and emotional health and they were more likely to perceive their current diet as harmful. Qualitative analyses found no consistent patterns among super-achievers in relation to some factors identified as important in previous studies, such as social support. However, super-achievers shared several characteristics such as determination and consistency in their engagement with the intervention, receptivity to new information and prompts, previous positive experience of changing health behaviours, being motivated by early success, making changes routine, and an ability to devise and apply strategies for dealing with setback and ‘relapse’ triggers.

**Conclusions:**

Successful lifestyle change depends on active engagement as well as effective intervention ingredients. Weight loss interventions are likely to be more effective where they can adapt to participants’ differing characteristics and needs, while also providing core elements likely to build success.

## Background

Prevention trials have shown that lifestyle interventions that achieve and maintain significant weight loss can have a favourable effect on diabetes and cardiovascular risk factors [1, 2]. It is plausible that these lifestyle changes would also have favourable effects on the risk of obesity-related neoplasia. With respect to colorectal cancer, Jacobs and colleagues [3] reported obesity as a risk factor for short-interval (3 year follow up) development of colorectal adenomas (identifiable pre-cursor lesions of CRC), and although it is unclear at what stage obesity impacts on adenoma development, there is evidence that adenoma risk increases amongst adults who have gained weight in the 5 years prior to colonoscopy investigations [4]. The same study also demonstrated that a higher BMI was a stronger risk factor for advanced adenoma recurrences, when compared with non-advanced lesions. It is interesting to note that a study from Japan of 1650 subjects diagnosed with an adenoma reported that the subsequent incidence of adenomas was significantly lower in people who lost weight in the one year follow up period than in those who maintained or gained weight [5]. The case for exploring weight reduction strategies in people diagnosed with CRC adenomas is also supported by long term follow up trials of bariatric surgery showing significant reductions in CRC risk [6].

In light of the evidence above, the colorectal cancer screening setting has been described as an unexplored opportunity for endorsing changes in health behaviours [7, 8]. The BeWEL randomised controlled trial (RCT) [9] demonstrated that the colorectal cancer screening setting offers a useful opportunity to initiate and achieve successful weight management in colorectal adenoma bearers aged 50 to 74 years with a body mass index (BMI) >25 kg/m^2^. The trial demonstrated sustained changes in body weight, physical activity, eating and drinking habits over a 12 month intervention programme delivered through three face-to-face visits with a counsellor and nine monthly telephone calls.

Both groups lost significant weight, with the intervention group losing on average 3.50 kg (SD 4.91 kg, 95 % CI 2.70 to 4.30) and the control group losing 0.78 kg (SD 3.77 kg, 95 % CI 0.19 to 1.38) [9]. However, the significant group difference of 2.69 kg (95 % CI 1.70 to 3.67) highlights the overall success associated with the intervention programme and merits further investigation to examine the differential responses of participants to the programme. For example, only 33 (22 %) participants achieved the programme target of >7 % body weight loss [9]. While there is increasing evidence that carefully designed programmes can lead to successful weight loss [10], the reasons for the variation in outcomes found in such programmes are not well understood.

A number of studies have attempted to shed some light on why some people are more successful at achieving or maintaining weight loss than others. For example, an online survey of participants in a commercial weight loss programme found that success was associated with various types of control over eating, such as greater dietary restraint, less tendency to eat to control mood and emotion, not skipping meals, not keeping snack foods in the house and eating takeaway foods less frequently [11]. Similarly, Fuglestad and colleagues’ [12] study of weight loss among individuals who had recently lost substantial weight on their own initiative found that greater regularity and control in eating was associated with greater recent weight loss and greater fruit and vegetable intake. However, neither study investigated the potential contribution of wider factors which might explain why some participants were better able to control their eating than others.

A systematic review [13] of the factors reported in quantitative studies which were associated with successful participation in lifestyle behaviour change programmes (not solely focused on weight loss) found that the factors most consistently associated with uptake of lifestyle change related to support from family and friends, transport and other costs, and beliefs about the causes of illness and lifestyle change, with depression and anxiety also appearing influential. However, the review reported that many factors showed inconsistent patterns with respect to uptake and completion of lifestyle change programmes. Another review, this time focusing on factors identified in qualitative studies [14], found that the most commonly reported influences were those relating to social support (whether provided formally or informally), beliefs (about the self or the causes and management of poor health, and the value of maintaining lifestyle behaviours), and other psychological factors (including attitude, thinking and coping styles, and problem solving skills). The same review notes that influencing factors are interlinked, and that while the literature may help us to identify individual factors associated with successful lifestyle change, there is still a limited understanding of the nature of the relationships between factors and how they differ between individuals. Similarly, Dalle Grave and colleagues [10] state that despite some gains in insight, knowledge regarding predictors of weight loss remains incomplete.

The current work aimed to explore the factors associated with success in weight loss in the BeWEL study by comparing, both quantitatively and qualitatively, those who succeeded in achieving more than 7 % weight loss with those who were less successful. The quantitative analysis examined the possible contribution of a range factors thought to be associated with weight loss, including socio-demographic and body weight characteristics, health perceptions, quality of life, self-efficacy and perceptions of lifestyle, while the qualitative study enabled a more open-ended exploration of possible factors.

## Methods

Quantitative and qualitative methods were used to identify the factors associated with success in weight loss. Two approaches were used: a sub-group analysis of quantitative data for those participants who participated in the BeWEL intervention and completed follow-up measures, and qualitative interviews with a sample of 24 intervention participants who achieved varying levels of weight loss success.

### Recruitment

Recruitment for the BeWEL RCT took place in four Scottish NHS health board areas from November 2010 to May 2013. Participants aged 50 to 74 years who had taken part in the Scottish Bowel Screening programme and undergone polypectomy for adenoma were informed about the study in writing. Those indicating an interest in participating were telephone-screened by a research nurse for eligible criteria: BMI >25 kg/m^2^, able to undertake physical activity and provide informed consent, not pregnant and without insulin dependent diabetes mellitus or any cancer diagnosis. Eligible participants were then posted a more detailed information leaflet and invited to attend their local study centre to provide informed written consent and undergo baseline measures.

### Baseline and follow up measures

Socio-demographic data on age, gender, ethnicity, marital status, education, employment and postcode were recorded at baseline [15]. Objective measures recorded at baseline, three months and 12 months included height, weight, waist circumference, blood pressure, fasting blood samples and physical activity. Physical activity levels were measured objectively using a SenseWear^TM^ armband (BodyMedia Inc. Pittsburgh, PA) worn on the upper arm for 7 days to provide participants’ daily step count, and time spent in sedentary, moderate (3 to <6 METS) and vigorous (≥6 METS) activities [16].

An interviewer-administered questionnaire was used to record self-reported dietary intake, frequency of alcohol consumption, and self-efficacy and perceptions of lifestyle [17, 18]. Participants were assigned scores for fibre, fat and unsaturated fat using the Dietary Instrument for Nutrition Education (DINE) questionnaire [19]. The fibre score (range 3–88) was based on the frequency of intake of bread, rice, potatoes, pasta, other starchy foods and fruit and vegetables (including beans and lentils). The fat score (range 7–>72) was based on intakes of substantial contributors to fat intake i.e. dairy foods, meat, processed meat, fish, fried foods, sweet and savoury snacks and fat spreads, and the unsaturated fat score (range 3–12) was based on the type of fats used.

Daily portions of fruit and vegetables were estimated using a modification of Cappuccio and colleagues’ two-item questionnaire [20], which asked ‘How many pieces of fruit and vegetables (excluding potatoes) do you eat—of any sort—on a typical day?’, recording fruit and vegetable portions separately. Fruit and vegetable juices only counted as a maximum of one portion per day. Portion sizes were illustrated using show-cards as defined by the NHS Livewell Portion Size guidance [21].

Sugary drink intake (excluding diet, low-calorie drinks and fresh fruit juice) was self-reported using nine frequency categories whereas typical consumption of alcohol (on week days and weekends) was assessed using questions from the Alcohol Use Disorders Inventory Test (AUDIT) [22] questionnaire.

Ethical approval was granted by NHS Tayside Committee on Medical Research Ethics (Ref 10/S1402/34).

### Randomisation

Following baseline measures, participants were randomised, 1:1, to parallel-groups using a permuted-block technique. Allocation was to either a control group (weight loss booklet) or a 12-month intervention group (three face-to-face visits with a counsellor and nine telephone calls, one per month).

### Intervention

The intervention protocol is described in full by Caswell and colleagues [23], but in brief targeted a 7 % reduction in body weight through diet, activity and behaviour change, including a personalised energy prescription (600kcals below that required for weight maintenance). Motivational interviewing techniques explored self-assessed confidence, ambivalence and personal values concerning weight. To assist change in both diet and physical activity, participants were encouraged to focus on one topic (diet or physical activity) during visit one, and the remaining topic on visit two, and were generally advised to begin with the topic where they had the strongest likelihood of success. Behaviour change techniques were employed including goal setting, identifying implementation intentions, self-monitoring of body weight (scales were provided) and counsellor feedback about reported diet, physical activity and weight change. The intervention was delivered in four NHS areas with one lifestyle counsellor in each area, and all counsellors received the same training to ensure consistency in delivery. The primary outcome was change in body weight at 12 months. Secondary outcomes included percentage weight loss, change in BMI and waist circumference, health behaviours (dietary and alcohol habits and physical activity) and self-reported psycho-social variables (self-efficacy and self-assessed health and quality of life).

### Sub-group analysis of quantitative data

A sub-group analysis was undertaken of the intervention participants who completed the study to compare participants according to their percentage weight loss. Participants were divided into ‘super-achievers’ (≥7 % weight loss), ‘moderate-achievers’ (2–< 7 % weight loss) or ‘low-achievers’ (<2 % loss, or weight gain). Weight loss across the sample was normally distributed. Descriptive statistics were used to quantify socio-demographic and body weight characteristics, health perceptions, quality of life, self-efficacy and perceptions of lifestyle at baseline, and changes in anthropometry and health behaviours (dietary and alcohol habits and physical activity) at 12 months. Chi-squared tests were used to identify differences in proportions, and analysis of variance with post-hoc Bonferroni tests (to minimise the likelihood of type 1 errors) to detect differences in continuous variables. Between group differences are expressed as Odds Ratios (OR) or means with 95 % Confidence Intervals (CI) and p-values (*p* < 0.05 signifies statistical significance).

Statistical analyses were carried out using SPSS (Version 21.0, IBM, Armonk, NY, USA).

### Qualitative interviews and analysis

Qualitative in-depth individual interviews were conducted with a sample of 24 intervention group participants on study completion, between July 2012 and January 2013. Purposeful sampling was employed, stratified by gender, socioeconomic status, and research site and weight loss achieved, with the aim of interviewing participants at all points on the weight loss spectrum. The achieved sample comprised almost equal proportions of males and females (13 and 12 respectively), and included participants across all five Scottish Index of Multiple Deprivation (SIMD) [24] quintiles. Baseline age, weight, BMI and waist circumference were representative of the whole cohort, although a higher proportion of the interviewees were classified as obese. Interviews were conducted in participants’ own homes and lasted an average of 60 min. Written informed consent was provided for participation in the interview and its audio-recording and transcription *verbatim*. An interview topic guide was developed which investigated the acceptability of the BeWEL programme, the participants’ initial expectations and motivations and the extent to which these were met by their subsequent experiences, and how well participants engaged with the programme. Factors influencing patients’ willingness and ability to comply with programme advice were explored including lifestyle, attitudinal and other differences which might explain variable response to and engagement with the programme.

All transcripts were imported into NVivo (Version 10, QSR International, Melbourne, Australia) to facilitate coding and analysis. The transcripts underwent several stages of analysis. Each transcript was read and coded by one of two researchers (JM and MS), with four transcripts read and coded by both researchers. Emerging themes were identified through a process of thorough familiarisation with the texts and discussion between the researchers. Drawing on the framework approach (eg. [25]), an initial framework was developed organised around the different aspects of engagement in the programme: the decision to participate and the factors associated with that decision, experiences of initial engagement in the programme, experiences of making changes, facilitators and barriers to change, and intentions and hopes for future maintenance. This framework was then expanded to incorporate more detailed examination of the motivators, facilitators and barriers to initiating and sustaining change. A series of matrices were generated which illustrated the pattern of factors for each participant. Using weight loss outcome data, the 24 participants were then grouped into ‘super-’, ‘moderate-’ and ‘low-achievers’ using the same percentage weight loss cut-offs as in the quantitative analyses. This resulted in 10 super-achievers, six moderate-achievers and eight low-achievers within the qualitative sample. The matrices of motivators, facilitators and barriers were then re-examined for any patterns relating to the three categories of weight loss. Pseudonyms are used throughout the paper to preserve participants’ anonymity.

## Results

### Quantitative study findings

Of the 148 (91 %) participants who were randomised to receive the BeWEL intervention and completed 12 month assessments, 33 (22 %) were ‘super-achievers’, 58 (39 %) ‘moderate-achievers’ and 57 (39 %) ‘low-achievers’.

Participants ranged in age from 50 to 75 years (reflecting the colorectal cancer screening age in Scotland). Overall, 74 % of the participants were male, and 35 % lived in the two most deprived SIMD quintiles of socio-economic deprivation. Almost half (*n* = 72, 49 %) were in the obese category (BMI >30 kg/m^2^) and 60 % (52 % males and 84 % females) reported having previously tried (80 % successfully) to lose weight (Table 1).

**Table 1:** Baseline socio-demographic and body weight characteristics of intervention participants who completed, by subsequent achievement category

	Super-achievers	Moderate-achievers	Low-achievers	Between group differences		*p*-value
				Mean or Odds Ratio (95%CI)		
	*n* = 33	*n* = 58	*n* = 57	Super vs. moderate	Super vs. low	
Male gender: n (%)	22 (66.7)	44 (75.9)	44 (77.2)	0.6 (0.2, 1.6)	0.6 (0.2, 1.5)	0.51
Age (years): Mean (SD)	65.2 (SD 6.7)	63.7 (SD 6.5)	62.3 (SD 7.2)	1.5 (−1.4, 4.3)	2.9 (−0.2, 5.9)	0.15
SIMD quintiles: n (%)						
1 (most deprived)	4 (12.1)	11 (19.0)	6 (10.5)	---	---	0.29
2	6 (18.2)	12 (20.7)	12 (21.1)			
3	3 (9.1)	13 (22.4)	10 (17.5)			
4	12 (36.4)	7 (12.1)	14 (24.6)			
5 (least deprived)	8 (24.2)	15 (25.9)	15 (26.3)			
In least deprived 5 SIMD deciles	21 (63.6)	29 (50.0)	37 (64.9)	1.8 (0.7, 4.2)	0.9 (0.4, 2.3)	0.22
Married/Cohabiting: n (%)	29 (87.9)	47 (81.0)	43 (75.4)	1.7 (0.5, 5.8)	2.4 (0.7, 7.9)	0.35
Retired: (%)	20 (60.6)	35 (60.3)	32 (56.1)	1.0 (0.4, 2.4)	1.2 (0.5, 2.9)	0.88
Baseline weight (kg): Mean (SD)	89.2 (12.1)	89.7 (16.1)	92.6 (15.5)	−0.4 (−6.8, 6.0)	−3.3 (−9.2, 2.5)	0.48
Baseline BMI (kg/m^2^): Mean (SD)	31.1 (3.4)	30.8 (4.9)	31.4 (4.9)	0.3 (−1.6, 2.2)	−0.3 (−2.0, 1.4)	0.78
Obese^a^ at baseline: n (%)	19 (57.6)	26 (44.8)	27 (47.4)	1.7 (0.7, 4.0)	1.5 (0.6, 3.6)	0.49
Baseline waist circumference (cm): Mean (SD)	105.5 (9.2)	103.5 (11.2)	106.4 (11.8)	2.0 (−2.5, 6.6)	−0.8 (−5.3, 3.6)	0.37
Made previous attempt at weight loss: n (%)	24 (72.7)	31 (53.4)	34 (59.6)	2.3 (0.9, 5.8)	1.8 (0.7, 4.6)	0.20
Successful at previous weight loss attempts: n (%)	21 (87.5)	25 (80.6)	30 (88.2)	1.7 (0.4, 7.5)	0.9 (0.2, 4.6)	0.65

^a^BMI > 30 kg/m^2^

When the three achievement groups were compared, no significant differences were found in their socio-demographic or body weight characteristics at baseline, nor their previous history of weight loss attempts (Table 1). In addition, no significant differences were found between NHS sites in baseline characteristics or weight loss outcomes, suggesting that ‘super-achievement’ was not explained by differences in implementation between the lifestyle counsellors allocated to each NHS site.

Following the 12 month intervention, ‘super-achievers’ had lost an average of 10.2 kg (11.5 %) body weight, 3.5 BMI units, and 11.7 cm from their waist circumference. In contrast, ‘moderate-achievers’ had lost 3.8 kg (4.2 %), 1.3 BMI units and 4.4 cm, and ‘low-achievers’ had gained 0.7 kg (0.8 %), 0.3 BMI units and lost 1.6 cm (Table 2).

**Table 2:** Behaviour changes associated with ‘super-achievement’ at 12 months

	Mean (Standard Deviation)			Between group differences		*P*-value
				Mean or Odds Ratio (95%CI)		
	Super-achievers	Moderate-achiever	Low-achiever	Super Vs Moderate	Super Vs Low	
	(*n* = 33)	(*n* = 58)	(*n* = 57)			
Anthropometry						
• Weight loss (kg)	−10.2 (4.3)	−3.8 (1.5)	0.7 (2.4)	−6.4 (−7.8,−5.0)	−11.0 (−12.4,−9.6)	<0.01*
• % body weight change	−11.5 (4.3)	−4.2 (1.4)	0.8 (2.6)	−7.2 (−8.7,−5.8)	−12.2 (−13.7,−10.8)	<0.01*
• BMI change (kg/m^2^)	−3.5 (1.5)	−1.3 (0.5)	0.3 (0.9)	−2.2 (−2.7,−1.7)	−3.8 (−4.3, 3.3)	<0.01*
• Change in waist circumference (cm)	−11.7 (5.1)	−4.4 (2.6)	−1.6 (3.9)	−7.3 (−9.3,−5.2)	−10.1 (−12.1,−8.1)	<0.01*
Physical activity						
• Change in daily average time spent active (mins)	25.2 (63.0)	−0.30 (68.9)	2.1 (43.9)	25.5 (−6.7, 57.6)	23.1 (−10.0, 56.2)	0.14
• Change in daily average time spent in:						
○ Sedentary activity (mins)	−29.8 (100.0)	−8.3 (187.0)	−23.6 (143.0)	−21.5 (−108.1, 65.2)	−6.2 (−94.6, 82.3)	0.80
○ Moderate activity (mins)	27.3 (61.8)	−1.0 (57.9)	1.9 (42.8)	28.3 (−1.6, 58.2)	25.4 (−5.2, 55.9)	0.06
○ Vigorous activity (mins)	0.9 (4.6)	−2.1 (18.5)	−0.7 (2.8)	3.0 (−3.8, 9.8)	1.6 (−5.4, 8.5)	0.56
• Change in daily average step count	1878 (3556)	−109 (3335)	−372 (2118)	1987 (363, 3611)	2250 (581, 3918)	<0.01*
Dietary intake						
• Change in fat consumption score	−8.6 (11.3)	−7.4 (8.2)	−6.6 (11.3)	−1.3 (−6.7, 4.2)	−2.0 (−7.5, 3.4)	0.66
• Change in unsaturated fat score	0.5 (1.7)	0.6 (1.8)	0.3 (1.5)	−0.1 (−1.0, 0.8)	0.2 (−0.7, 1.1)	0.59
• Change in fibre food consumption score	0.5 (9.0)	0.6 (10.6)	−1.6 (11.1)	−0.8 (−5.6, 5.4)	2.1 (−3.4, 7.7)	0.47
• Increased fruit and vegetable portions (%)	25 (75.8 %)	32 (55.2 %)	20 (35.1 %)	2.54 (0.98, 6.56)	5.78 (2.20, 15.16)	<0.01*
• Lowered sugary drink consumption [drinkers only] (%)	5 (71.4 %)	10 (90.9 %)	3 (60 %)	0.25 (0.02, 3.47)	1.67 (0.15, 18.9)	0.33
Lowered alcohol consumption [drinkers only] (%):						
• Frequency	5 (19.2 %)	19 (37.3 %)	14 (27.5 %)	0.40 (0.13, 1.24)	0.63 (0.20, 1.99)	0.24
• Amount on weekdays	9 (34.6 %)	15 (29.4 %)	14 (27.5 %)	1.27 (0.46, 3.48)	1.40 (0.51, 3.86)	0.54
• Amount at the weekend	4 (15.4 %)	10 (19.6 %)	13 (25.5 %)	0.75 (0.21, 2.65)	0.53 (0.15, 1.83)	0.43

Chi-squared test used for differences in proportions, Independent samples *t*-test used for differences in continuous variables

^*^ *p* < 0.05, significant

Lifestyle behaviours assessed at the end of the 12 month intervention period indicated that super-achievers had increased their daily step count by 1878 ± 3556 steps per day, whereas moderate and low-achievers had reduced theirs (−109 ± 3335 and−372 ± 2118 steps per day, respectively, *p* < 0.01). A higher proportion of super-achievers also increased the number of portions of fruit and vegetables they reported consuming per day, but no other differences in fat or fibre scores, or in the proportions reducing their consumption of sugary drinks or alcohol between the groups, were identified.

The majority (79 %) of participants had rated their current health as ‘good’ or better at baseline. However, while there were no significant differences in perceptions of current health, super-achievers were significantly less likely than the rest to report that their activities (e.g. stair climbing, moderate activity and general accomplishments) and work were affected by physical and emotional health (Table 3).

**Table 3:** Baseline health perceptions and quality of life of intervention participants who completed, by subsequent achievement category

				Between group differences		*p*-value
				Odds Ratio (95%CI)		
	Super-achievers	Moderate-achievers	Low-achievers	Super vs. moderate	Super vs. low	
	*n* = 33	*n* = 58	*n* = 57			
Health poor/fair: n (%)	2 (6.1)	12 (20.7)	20 (35.1)	0.2 (0.1, 1.2)	0.2 (0.0, 0.6)	0.06
Health limits moderate activities: n (%)	3 (9.1)	11 (19.0)	19 (33.3)	0.4 (0.1, 1.7)	0.2 (0.1, 0.7)	0.02*
Health limits ability to climb stairs: n (%)	6 (18.2)	13 (22.4)	28 (49.1)	0.8 (0.3, 2.3)	0.2 (0.1, 0.6)*	<0.01*
Accomplishes less due to physical health: n (%)	4 (12.1)	14 (24.1)	22 (38.6)	0.4 (0.1, 1.4)	0.2 (0.1, 0.7)*	0.02*
Limited due to physical health most/all of the time: n (%)	1 (3.0)	3 (5.2)	8 (14.0)	0.6 (0.1, 5.7)	0.2 (0.0, 1.6)	0.11
Accomplishes less due to emotional health: n (%)	5 (15.2)	18 (31.0)	18 (31.6)	0.4 (0.1, 1.2)	0.4 (0.1, 1.2)	0.19
Work less carefully due to emotional health: n (%)	0 (0)	11 (19.0)	11 (19.3)	---	---	0.03*
Pain interferes with normal work; n (%)	11 (33.3)	24 (41.4)	30 (52.6)	0.7 (0.3, 1.7)	0.5 (0.2, 1.1)	0.18
Feel calm/peaceful most/all of the time: n (%)	23 (69.7)	33 (56.9)	40 (70.2)	1.7 (0.7, 4.3)	1.0 (0.4, 2.5)	0.27
Feel full of energy most/all of the time: n (%)	19 (57.6)	27 (46.6)	23 (40.4)	1.6 (0.7, 3.7)	2.0 (0.8, 4.8)	0.29
Feel depressed most/all of the time: n (%)	0 (0)	5 (8.6)	4 (7.0)	---	---	0.24
Problems interfered with social activities most/all of the time: n (%)	3 (9.1)	4 (6.9)	4 (7.0)	1.4 (0.3, 6.4)	1.3 (0.3, 6.3)	0.92

^*^ *P* < 0.05, significant

Super-achievers were no different in how they had rated their self-efficacy, beliefs about their lifestyle risk and perceptions of their own lifestyle and bodyweight at baseline, with one exception: they were significantly more likely to believe their diet was harmful to their health (Table 4).

**Table 4:** Baseline self-efficacy and perceptions of lifestyle in intervention participants who completed, by subsequent achievement category

	Mean (Standard Deviation) rating: (range 1 = lowest, 7 = highest)			Between group differences		*p*-value
				Mean (95%CI)		
	Super-achievers	Moderate-achiever	Low-achiever	Super vs. Moderate	Super vs. Low	
	(*n* = 33)	(*n* = 58)	(*n* = 57)			
Self-efficacy ^a^						
• How sure are you that you will control your weight?	5.0 (1.3)	5.0 (1.8)	5.1 (1.7)	0.00 (−0.88, 0.88)	−0.14 (−1.0, 0.74)	0.88
• How sure are you that you will exercise regularly?	5.6 (1.5)	5.7 (1.5)	5.4 (1.8)	−0.16 (−1.01, 0.69)	0.20 (−0.66, 1.04)	0.49
• How sure are you that you will control your diet?	5.2 (1.1)	5.4 (1.4)	5.3 (1.5)	−0.15 (−0.88, 0.58)	−0.08 (−0.83, 0.66)	0.88
• How sure are you that you will control alcohol intake?	6.0 (1.3)	6.0 (1.5)	5.8 (1.5)	0.03 (−0.74, 0.80)	0.22 (−0.56, 0.99)	0.73
Perceptions of risk^b^						
• Do you believe being overweight is harmful?	6.7 (0.6)	6.4 (1.0)	6.5 (0.7)	0.27 (−0.16, 0.70)	0.12 (−0.31, 0.56)	0.30
• Do you believe not exercising regularly is harmful?	6.3 (1.0)	6.0 (1.5)	6.4 (0.8)	0.27 (−0.34, 0.88)	−0.15 (−0.76, 0.46)	0.15
• Do you believe your current diet is harmful?	4.0 (1.7)	3.0 (1.7)	3.8 (1.7)	1.03 (0.13, 1.93)*	0.27 (−0.64, 1.18)	0.01*
• Do you believe that excessive alcohol intake is harmful?	6.8 (0.5)	6.8 (0.7)	6.6 (1.3)	−0.04 (−0.53, 0.46)	0.19 (−0.31, 0.69)	0.39
Perceptions of own lifestyle and body weight						
• How would you describe your present bodyweight?^c^	5.7 (1.0)	5.3 (1.1)	5.3 (1.2)	0.46 (−0.14, 1.07)	0.47 (−0.14, 1.08)	0.12
• How would you describe your typical alcohol intake?^d^	3.0 (1.7)	3.3 (1.7)	3.3 (1.7)	−0.28 (−1.17, 0.62)	−0.31 (−1.21, 0.60)	0.68
• How do you rate your weekly physical activity levels?^e^	3.7 (1.3)	4.0 (1.4)	3.7 (1.6)	−0.31 (−1.08, 0.47)	0.02 (−0.76, 0.80)	0.43
• How would you describe your red meat intake?^f^	3.6 (1.4)	3.4 (1.2)	3.8 (1.4)	0.14 (−0.57, 0.84)	−0.24 (−0.95, 0.47)	0.32
• How would you describe your soft drink intake?^f^	2.5 (1.7)	2.3 (1.5)	2.0 (1.1)	0.22 (−0.54, 0.98)	0.50 (−0.27, 1.27)	0.27
• How would you describe your water intake?^f^	3.4 (1.5)	3.7 (1.6)	3.6 (1.7)	−0.30 (−1.16, 0.55)	−0.23 (−1.10, 0.64)	0.69

*p < 0.05, significant

^a^1 = Not at all sure, 7 = Very sure

^b^1 = Not at all harmful, 7 = Very harmful

^c^1 = I am very underweight, 7 = I am very overweight

^d^1 = I don’t drink alcohol, 7 = I drink far too much alcohol

^e^1 = I do no activity, 7 = I am far too active

^f^1 = I do not eat/drink, 7 = I eat/drink far too much

### Qualitative study findings

Analysis across the sample, comparing super, moderate and low-achievers, found no clear patterns in relation to some key factors which previous studies have suggested may contribute to differences in success, such as family and social support, quality of relationship with counsellor and access to healthy food and opportunities for physical activity. Although having an involved and supportive spouse was important to some super-achievers, others, particularly female super-achievers, did well with either no partner to support them, or in the context of family indifference to their efforts. Similarly, although participants generally spoke positively about the counsellors, the depth and nature of the relationship varied, and there appeared not to be a consistent link between degree of weight loss achievement and relationship with the counsellor: for example, one super-achiever had requested extra contact with the counsellor during the 9-month telephone support period because he felt he needed this external prompting and validation to keep on track, while another super-achiever could not even remember the counsellor’s name, and was satisfied with a much more arm’s length relationship. What seemed more important was that the individual was sufficiently driven to make changes for themselves and did not feel the need for external approbation from those around them:

“Once you are into it it’s your own discipline, it doesn’t matter what they say to you because in the end it’s up to you, they can encourage you and everything else but they can’t make you do anything … it’s up to yourself, totally up to yourself. They are only there for suggestions and encouragement, that’s all” (‘Joe’, aged 70, super-achiever).

In terms of access to healthy food and opportunities for physical activity, participants generally felt that they had been able to afford any changes to their shopping patterns, albeit that prices had risen generally over the study period because of the economic recession. Some low-achievers offered reasons for their lack of physical activity based on unsuitable facilities—for example, that a local swimming pool was not set up for lane swimming—but these appeared to mask underlying reasons such as a dislike of exercise. Many observed that walking, the main physical activity change made in most cases, did not cost anything.

Whilst the themes of support and access did not appear to help explain differences in achievement, several patterns did emerge in relation to other themes both across the sample and from looking in-depth at the super-achiever sample. These were health, lifestyle adaptation, flexibility, drawing on previous success, coping with set-backs, motivation, determination/commitment, and the use of prompts, aids and information.

#### Health

Several participants experienced health problems which made it difficult for them to undertake exercise. One low-achiever, ‘Len’, aged 64, described how a stroke, back problems and a history of work-related injuries had left him tremendously frustrated at his inability to be as active as he once was; he felt that these physical problems had been an important barrier to success. The pattern across the sample was not clear-cut, with not all low-achievers having health problems and some super-achievers facing substantial health challenges—‘Bill’, aged 62, for example, managed to lose 9.7 kg despite sciatica, thyroid problems, incontinence, pain in his legs and spine which made walking difficult, and mental health problems. However, there was a tendency for more importance to be attached to physical and mental health problems by low-achievers than by super-achievers in their accounts of how and why they had progressed on the programme.

#### Lifestyle adaptation

Super-achievers tended to describe having made specific and extensive changes, both to diet and to physical activity, and to have incorporated these changes quickly into their daily routines. For example, several adopted daily walking, even in bad weather, or other forms of exercise. Many also reported concerted efforts to reduce their intake of high fat foods and processed meats; to increase their intake of fruit and vegetables, low fat variants and fibre; and to control their portion sizes and frequency of eating throughout the day. Low-achievers, in contrast, tended to talk more vaguely about the changes they had attempted, with references to “being more aware of” what they should be doing, “trying to” change and “making little tweaks”, but fewer specific examples. Some felt they were already doing the right things or doing as much as they could, while others admitted that they struggled against the temptation to “overload your plate” (‘Duncan’, aged 65, low-achiever) or to snack: “It’s the portions. I have to try and regulate portions. Also I have to stop mooching in the fridge at night—boredom” (‘Hetty’, aged 72, low-achiever).

#### Flexibility

Although super-achievers made these extensive changes to diet and physical activity, they permitted themselves some flexibility within this wider framework of change. One strategy was not to waste energy on attempting changes to which they knew they were not committed: as ‘Joe’, aged 70, put it, “Brown rice, I’d rather jump out the window than eat brown rice”. Another strategy was to permit occasional lapses and treats, not regarding them as taboo or as signs of failure, but as permissible so long as the overall direction of change remained positive. Indeed, several super-achievers felt they could not have been successful if they had adhered to a joyless regime in which these ‘treats in moderation’ were not permitted.

#### Drawing on previous success

For super-achievers (and to a lesser extent some of the moderate-achievers), early experience of weight loss within BeWEL drove them on—“I couldn’t believe how it was coming off, just going out [for] walks” (‘Sandra’, aged 65, super-achiever); “I liked the fact that when I would go on the scales on a weekly basis and I had maybe just lost that wee bit, the thrill it gave me. Oh you dancer! [expression of pleasure]” (‘Ronnie’, aged 69, moderate-achiever). Super-achievers could also draw on successful change which they had achieved prior to BeWEL, such as having quit smoking, to bolster their determination and confidence: “I was really being very strong willed at that point in time. I’d mastered the smoking which had really—I thought if I can do the smoking, I can definitely get rid of the weight” (‘Aggie’, aged 67, super-achiever).

#### Coping with set-backs

Setbacks, such as a plateauing of weight or weight gain, were experienced by many participants. Here, a key difference between super-achievers and low and moderate-achievers was in how these were dealt with. Low-achievers in particular tended to become demoralised by poor progress or by relapses, leading to a weakening of subsequent effort: ‘Eileen’, aged 63, described it as “a wee bit soul destroying after about 3 weeks and you’ve only lost a pound or something”, while ‘Len’, aged 64, reflected that his impatient nature had led to feelings of frustration when progress seemed slow or non-existent: “If things don’t happen quickly enough for me—that is just cos of the way I’ve been all my life—I like to get on and get things done. And I just wonder if that [was what made] a difference”. Super-achievers were similarly “disappointed” or “annoyed” when their weight loss progress appeared to stall or reverse, but, importantly, were spurred on by these feelings, and by the memory of earlier success, to renew their efforts.

#### Motivation, determination and commitment

These were key themes in the interviews. Super-achievers made frequent reference to their own “determination” and “discipline” and identified these as key factors in their persistence and ultimate success. Several invoked notions of ‘doing themselves justice’ by committing wholeheartedly to the study and seeing it through:

“Just willpower and thinking I’m not letting [the counsellor] down and the nurse. …

It’s not that, if I let Karen and the nurse down I was letting myself down as well and I didn’t want to do that either” (‘Bill’, aged 62, super-achiever).

“There was a sense that you had a responsibility to do it honestly.” (‘Joe’, aged 70, super-achiever).

Similarly, some low-achievers identified that, while their initial expectations and motivation had been high, their willpower had been lacking and had let them down:

“At the end of the day, it was me who didn’t stop eating the ice cream and it was me who didn’t do the exercise, so I can only blame myself” (‘George’, aged 72, low-achiever).

#### Prompts, aids and information

Finally, for some super-achievers, simple prompts, aids and information proved helpful in motivating and sustaining change. For example, ‘Aggie’ responded well to the simple provision of scales (she had not weighed herself in years) and a pedometer, which gave her a daily target. ‘Joe’ in contrast felt no further need of the pedometer after using it for a few days and did not want any of the exercise aids offered, but became fascinated by information about the calorie content of foods, a subject he had never previously considered, and drew on this new knowledge to reinforce his dietary changes. For ‘Ken’, completing the sheets and forms offered by the counsellor for recording daily time spent in physical activity became a project he completed “religiously” and with great enjoyment. It is likely that these prompts, aids and pieces of information would not have been sufficient in themselves to motivate and sustain changes without the other factors discussed above, but in conjunction with them, for some super-achievers they were helpful.

## Discussion

During a 12 month weight loss programme, one group of participants (‘super-achievers) attained significantly greater reductions in body weight, BMI and waist circumference compared to other achievement groups. These changes were accompanied by some improvements in both diet and physical activity. Whilst the data did not indicate any differences in demographic characteristics between achievement groups, significantly fewer super-achievers reported that their activities were limited by their physical (and emotional) health compared to those who lost less weight, and super-achievers were more likely to think that their current diet was harmful. Differences in achievement were not explained by differences in intervention delivery: no significant differences were found between participants in different areas in baseline characteristics or weight loss outcomes, suggesting that greater achievement by some participants was not related to differences in the type of support they received. Qualitative analysis showed that super-achievers shared several characteristics such as determination and consistency in their engagement with the intervention, receptivity to new information and prompts, previous positive experience of changing health behaviours, being motivated by early success, making changes routine, and an ability to devise and apply strategies for dealing with setback and ‘relapse’ triggers. Both the quantitative and the qualitative analysis suggested that physical and mental health problems were a possible contributory factor to the lower levels of engagement and success among moderate and low-achievers. These finding serve as a reminder of the challenge of addressing low levels of physical activity amongst multi-morbid populations in Scotland where it has been estimated that in areas of deprivation, by age 50, half of all Scots have at least one morbidity and by age 65 most are multi-morbid [26].

One limitation of this study is that the participants are likely to represent a somewhat motivated group, having voluntarily taken part in both the screening process and the subsequent weight loss trial. Uptake of bowel screening is also known to be poorer in more socio-economically deprived groups [27], and this is to some extent reflected in the distribution of participants in the quantitative analysis. However, deprivation had no influence on weight loss achievement, and the relatively even distribution of participants throughout qualitative analysis ensured the views of those at all levels of deprivation were represented. Consideration should also be given to the fact that this analysis purposefully considered only those in the intervention arm in an effort to pinpoint aspects of the intervention that were most or least effective. While this could have limited our understanding of the impact of any non-interventional factors on weight loss, considerable opportunity was given in the interview topic guide for participants to discuss these. It should also be noted that the BeWEL trial sample size was based on providing sufficient power (80 %) to detect a 7 % weight loss, and not on the ability to detect the differences in the sub-group comparisons reported here. Therefore, the quantitative results should be interpreted on their basis of being able to provide indicative findings that may warrant further testing in a larger fully powered trial.

A particular strength of this study was the combination of data collection methods, which combined both quantitative and qualitative analyses. While the quantitative analysis established the relative importance (or not) of various demographic, health status and attitudinal measures, the qualitative analysis was able to explore in more depth the differences in mindset and approach which appeared to characterise the super-achievers. This analysis suggested that success appeared to be associated with engaging in the study wholeheartedly (in particular, making changes routine and adhering to them) and taking personal responsibility for change rather than blaming external factors for any lapses in willpower. Successful participants also drew on new information, such as information on calorie content or number of steps walked per day, to motivate and reinforce determination. These findings are consistent with Rise and colleagues’ [28] qualitative analysis of factors associated with successful lifestyle change in a type 2 diabetes programme, which identified ‘new knowledge’, ‘taking responsibility’ and ‘formation of new habits’ as some of the important factors. It was also notable that, in the quantitative analysis, super-achievers were more likely at baseline to perceive their current diet to be ‘harmful’. Being willing to acknowledge personal inadequacies in their diet may have motivated them to identify specific areas for improvement.

The findings in the current study also echo some of the themes described in a qualitative study [29] with Scottish adults, where weight maintenance was associated with a range of behavioural strategies and making several (typically small) adjustments to diet and physical activity. The same study also found that those who successfully maintained their weight used more cognitive strategies for coping with setbacks and tended not to worry about minor fluctuations in weight or lapses in lifestyle habits, while in contrast, those who gained weight tended to give up at signs of difficulty or perceived failure. Similar themes emerged in the BeWEL study, with super-achievers tending to be committed to weight loss but also being flexible rather than fanatical in their adherence to the new regime, such that they could incorporate occasional treats and setbacks without losing focus or motivation and thus avoiding an ‘all or nothing’ approach which may lead people to attempt unrealistic goals [30, 31]. Another factor which did help super-achievers was being able to draw on previous experience of success, such as having given up smoking before entering the BeWEL study, or experiencing early weight loss in the first few weeks of the study. These experiences gave participants the knowledge and confidence that they could meet their weight goals and reinforced their motivation.

Interestingly, the qualitative analysis did not suggest that partner/family and social support helped to explain the differences in weight loss between super-achievers and others. This is a departure from the findings of several other studies [13, 14, 28, 32], which posit social support as an important factor in successful behaviour change. In the current study, super-achievers—several of whom lived on their own or engaged in the programme against a backdrop of family indifference—appeared to draw as much on their own reserves and motivation as on the support of those around them. While this is not to deny the importance of social support in lifestyle change programmes, it does suggest that it is not a pre-requisite for success. This is an encouraging finding in relation to people who engage in lifestyle change programmes with limited social support.

It has become an established approach to seek to identify the intervention factors associated with effectiveness [33, 34]. The rationale is that, if effective intervention ingredients can be identified and implemented, impact is likely to be greater. BeWEL used intervention techniques which are associated with greater effectiveness, such as goal-setting, self-monitoring, and providing feedback [35]. However, interventions are not one-way channels, through which effective ingredients are poured on to recipients; interventions only ‘work’ when people engage with and act on them. What was clear from the BeWEL study was that even where lifestyle counsellors used the same set of methods and techniques with each individual, some responded more successfully than others, for reasons to do with motivation, previous experience, willpower, ways of thinking about the challenge and other issues. The BeWEL findings highlight the need not to lose sight of the person at the centre of the intervention. Greaves and colleagues [35] underline this point in their review of facilitating factors for obesity-reducing lifestyle change, noting that people have different needs both from each other and at different moments in time. This highlights the importance of offering opportunities and support for lifestyle change not just on a one-off basis in a single intervention, but at multiple points over the life course and at all key encounters with health services, so that people can engage with them opportunely when motivation and circumstances coincide.

## Conclusions

The findings point to several implications for future weight loss interventions. Support and interventions for weight loss are likely to be more effective where they are able to adapt flexibly to participants’ differing characteristics and needs, while at the same time providing core elements likely to build success. These core elements include: providing early experience of success (to encourage continued effort and commitment); understanding what prompts might be motivating to particular individuals at different points in the process; helping participants to identify and make changes which are quickly incorporated into daily routines, whilst at the same time encouraging them to adopt flexible thinking which can cope with setbacks and lapses; and helping participants to identify previous experience of success which they can draw on to motivate and sustain them.
